# An Evaluation of Suitable Habitats for Amur Tigers (*Panthera tigris altaica*) in Northeastern China Based on the Random Forest Model

**DOI:** 10.3390/biology12111444

**Published:** 2023-11-17

**Authors:** Chunyu Gao, Yang Hong, Shiquan Sun, Ning Zhang, Xinxin Liu, Zheyu Wang, Shaochun Zhou, Minghai Zhang

**Affiliations:** 1 College of Wildlife and Protected Area, Northeast Forestry University, Harbin 150040, China; 13251612608@163.com (C.G.); hy1624@126.com (Y.H.); ssq2504204784@163.com (S.S.); 18846797329@163.com (N.Z.); z3o1415926@163.com (X.L.); wangzyu226@163.com (Z.W.); 2 Heilongjiang Wildlife Research Institute, Harbin 150081, China; zhoushaochun2003@163.com

**Keywords:** Amur tiger, habitat, prey potential richness, random forest model

## Abstract

**Simple Summary:**

Using the random forest model to predict the potential habitat of Amur tigers (*Panthera tigris altaica*) in Heilongjiang Province and Jilin Province of northeastern China was based on animal occurrence sites and various environmental variables. The results show that the suitable habitat for the Amur tiger was mainly distributed in the southern part of the Laoyeling Mountains (Hunchun–Wangqing–Dongning–Suiyang) at the border between Jilin and Heilongjiang provinces, the Sino-Russian areas of the border of Huilin–Raohe in the eastern part of the Wanda Mountains, and Yichun Forest Areas in the Lesser Khingan Mountains. The potential habitat of Amur tigers in the study areas was small and severely fragmented, with a lack of connectivity between patches. The results of this study suggested that habitat protection, restoration, and ecological corridor construction should be strengthened to increase population dispersal and exchange, providing technical support for the Amur tiger population conservation and habitat restoration in the future.

**Abstract:**

Amur tigers are at the top of the food chain and play an important role in maintaining the health of forest ecosystems. Scientific and detailed assessment of the habitat quality of Amur tigers in China is the key to maintaining the forest ecosystem and also addressing the urgent need to protect and restore the wild population of Amur tigers in China. This study used the random forest method to predict the potential habitat of Amur tigers in Heilongjiang and Jilin provinces using animal occurrence sites and a variety of environmental variables. Random forests are a combination of tree predictors such that each tree depends on the values of a random vector sampled independently and with the same distribution for all trees in the forest. The generalization error for forests converges to a limit as the number of trees in the forest becomes large. The generalization error of a forest of tree classifiers depends on the strength of the individual trees in the forest and the correlation between them. The results showed that the AUC value of the test set was 0.955. The true skill statistic (TSS) value is 0.5924, indicating that the model had good prediction accuracy. Using the optimal threshold determined by the Youden index as the cutoff value, we found that the suitable habitat for Amur tigers in the field was approximately 107,600 km^2^, accounting for 16.3% of the total study areas. It was mainly distributed in the Sino-Russian border areas in the south of the Laoyeling Mountains at the junction of Jilin and Heilongjiang provinces, the Sino-Russian border areas of Hulin–Raohe in the eastern part of the Wanda Mountains, and the Lesser Khingan Mountain forest region. The habitat suitability of the Greater Khingan Mountain and the plain areas connecting Harbin and Changchun was relatively low. Prey potential richness was the most critical factor driving the distribution of Amur tigers. Compared with their prey, the potential habitats for Amur tigers in Heilongjiang and Jilin provinces were small in total areas, sporadically distributed, and had low continuity and a lack of connectivity between patches. This indicates that some factors may restrict the diffusion of the Amur tiger, whereas the diffusion of ungulates is less restricted. The Amur tigers in this area face a serious threat of habitat fragmentation, suggesting that habitat protection, restoration, and ecological corridor construction should be strengthened to increase population dispersal and exchange. We provide a reference for future population conservation, habitat restoration, construction of ecological migration corridors, and population exchange of Amur tigers.

## 1. Introduction

Habitats are crucial for the survival and reproduction of wildlife, and high-quality habitats can improve survival and reproduction rates [1]. Therefore, the identification and evaluation of habitats are important links in wildlife conservation. The sustainable survival of large cats requires large areas of interconnected high-quality habitats [2]. The Amur tiger (*Panthera tigris altaica*), one of the most endangered large feline species in the world, is at the top of the food chain and plays an irreplaceable role in maintaining forest ecosystem health. However, with the development of society and economy, human construction and interference activities continue to increase. This has resulted in a sharp reduction in the forest areas in Northeast China, causing the habitat of the Amur tiger to shrink and become fragmented [2]. High-quality and safe habitats are important for the restoration of the Chinese Amur tiger population. Scientific diagnosis and habitat evaluation are the primary prerequisites for improving habitat quality, promoting the restoration of the wild tiger population, and maintaining their long-term survival and reproduction.

Prey potential richness is a key factor limiting the distribution and quantity of Amur tigers, as well as a key parameter defining their habitat [3]. Adequate prey resources are the basis and prerequisite for effective recovery and long-term survival of the Amur tiger population [4,5]. Specific thresholds exist for the food supply of ungulates to habitats and the supply of carnivores to ungulates. Prey and predator populations exhibit exponential growth and rapid recovery opportunities only when the resources in the habitat exceed a specific ecological threshold range [6]. The prey resources of the Amur tiger mainly include ungulates, such as Sika deer (*Cervus nippon*), red deer (*Cervus elaphus*), Siberian roe deer (*Capreolus pygargus*), and wild boar (*Sus scrofa*) [7]. An accurate assessment of the population resources of Amur tiger prey is the foundation for conducting habitat assessments of Amur tigers. Early methods for evaluating prey resources were mainly the line transect method, which is mainly applied to wildlife surveys [8]. With the development of infrared camera technology, the evaluation of wild Amur tiger resources using infrared cameras has gradually become one of the main methods of investigation [9,10]. Infrared cameras can be used to identify individuals based on the side patterns of tigers to obtain an accurate number of tigers in the monitoring area. Ungulate animals do not have patterns similar to those of tigers, making individual identification difficult. Consequently, it is challenging to directly obtain the number or density of prey in an area [11]. However, researchers have developed spatially explicit capture–recapture procedures (SECR) and other related models to evaluate the density of tiger prey through factors such as prey location, habitat, and time captured by infrared cameras. These models can also effectively estimate prey density and have been widely used [12,13].

Species distribution models (SDMs) use known species distribution points and environmental data as inputs and use mathematical, statistical methods or machine learning theory to explore the actual situation of species in niche space [14]. Species distribution models have been developed based on niche theory, mathematical statistics, and machine learning [15]. These models use computer algorithms to predict species data based on their geographical spatial distribution, which is a mathematical representation of their known distribution in environmental space [16]. Different models are suitable for different species distribution data. If there are only data on the occurrence points of species, methods based on envelope surface and distance analysis can be applied. If there are data on occurrence points–nonoccurrence points or occurrence points–pseudo nonoccurrence points, then regression methods and machine learning methods can be applied [2]. Machine learning methods perform well in predicting the models. Elith et al. (2006) [17] used 11 different methods to simulate the distribution of 226 plants and found that the maximum entropy model MaxEnt had the best average prediction ability. Other studies have also pointed out that machine learning methods such as boosted regression tree (BRT) [18] and random forest (RF) models have shown better performance [19,20]. The Maximum entropy model (MaxEnt Model) is based on the maximum entropy theory and was used and compared with other models by Elith et al. for species habitat modeling [17]. Its drawback is that it is very sensitive to sampling bias and is prone to overfitting. Random forest (RF), proposed by Breiman in 2001 [21], is currently one of the most widely used machine-learning algorithms. Owing to their insensitivity to multicollinearity, good tolerance for outliers and noise, and strong robustness, random forests have been applied in multiple fields [22]. It performs well in classification models (i.e., classification target variables) and can be used in regression models (i.e., continuous target variables). We based our research on a random forest model using biodiversity observation data with geographic coordinate references and corresponding habitat information as layers to predict species distribution. Predicting suitable habitats for Amur tigers in the whole distribution areas of China (both Heilongjiang Province and Jilin Province) is crucial for guiding the protection of wild populations of Amur tigers, their habitats, and forest ecosystems in Northeast China. This study assumes that there is a correlation between the observed data of species and their corresponding habitat information and uses mathematical models to output relatively reliable prediction maps.

## 2. Study Areas and Methods

### 2.1. Study Areas

**Figure 1:**
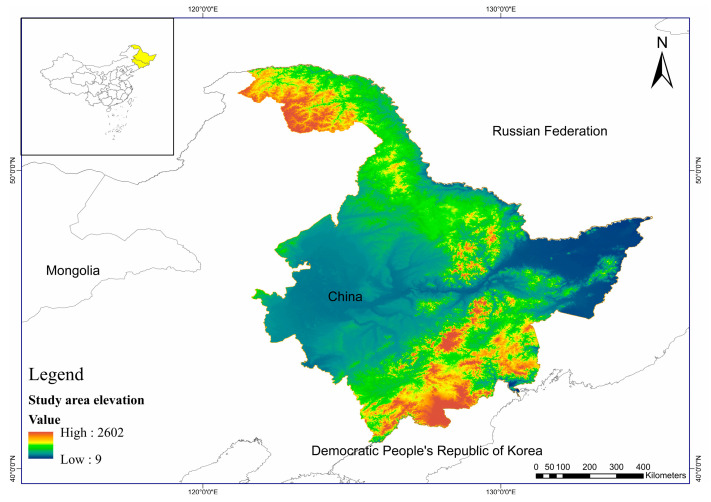
Geographical location of the study areas. Source: GEBCO Compilation Group (2023) GEBCO 2023 Grid (https://doi.org/10.5285/f98b053b-0cbc-6c23-e053-6c86abc0af7b, accessed on 5 January 2023).

At present, wild Amur tigers are only distributed in the eastern forest areas of Heilongjiang Province and Jilin Province in the northeast region, with geographical coordinates ranging from 121°11′ to 135°05′ east longitude to 43°26′ to 53°33′ north latitude (Figure 1). The eastern and northern regions are bordered by Russia by the Ussuri and the Heilongjiang Rivers, respectively. It borders North Korea to the east, Liaoning Province to the south, and Inner Mongolia Autonomous Region to the north. The study areas are surrounded by the Heilongjiang, Wusuli, Tumen, and Wusuli Rivers to the east and north, with abundant water resources. The west, north, and east of the inner areas are surrounded by high, medium, and low mountains and hills. These are encompassed by the Greater Khingan Mountains, Lesser Khingan Mountains, and the Changbai Mountains. The central part is the Songliao Plain, and the northeast part is the Sanjiang Plain. The altitude ranges from 34 m to 2691 m. The terrain of the entire area is relatively flat, consisting mainly of plains and hilly terrain. The study areas span between the temperate and cold temperate zones from south to north and have a temperate monsoon climate with four distinct seasons. It is warm and rainy in summer and cold and dry in winter. From southeast to northwest, the annual precipitation decreases from 1000 mm to below 300 mm. The Lesser Khingan and Greater Khingan Mountains are distributed in cold temperate coniferous and broad-leaved forests. The Greater Khingan Mountains are the southernmost part of the northern coniferous forests in Eurasia and belong to the southern part of the eastern Amur deciduous coniferous forests, which extend southward along the mountain range. The elevation of the Greater Khingan Mountains is approximately 600 m–1000 m, with a distribution of frozen soil layers. The zonal vegetation is the deciduous pine forest, with an obvious vertical zoning phenomenon. The temperate northern coniferous broad-leaved mixed forest zone includes the Lesser Khingan Mountains, Wanda Mountains–Zhangguangcai Mountains, and Muling–Sanjiang Plain. The temperate southern coniferous broad-leaved mixed forest zone includes the Changbai Mountain areas [23].

There is a wide variety of plants in this region, with approximately 2500 species of vascular plants. From the perspective of flora, it belongs to the “Changbai Flora” and is the central part of the flora. The majority of the study areas are located in the Palearctic realm, and there are also a few species in the Oriental realm that extend and are distributed, belonging to the northeastern region of China’s zoogeographical division. The climate in these areas is cold, with the Lesser Khingan and Greater Khingan Mountains staying below 0 °C for more than half a year and dense forests. Therefore, forest animals with cold tolerance in these areas are abundant, and the carnivorous order of mammals mainly includes the Amur tiger, Amur leopard (*Panthera pardus orientalis*), and Asian black bear (*Ursus thibetanus*). Forested areas have abundant plant-based foods and good hiding conditions, which are conducive to the survival of forest-dwelling animals such as ungulates. Among them, there are ungulates, such as the Siberian roe deer, red deer, moose (*Alces alces*), and wild boar. There are the most diverse bird species among the resident birds, including grouse and pheasant families. Reptiles and amphibians are relatively scarce in these areas.

### 2.2. Methods

#### 2.2.1. Prediction of Prey Potential Richness in Amur Tigers

**Figure 2:**
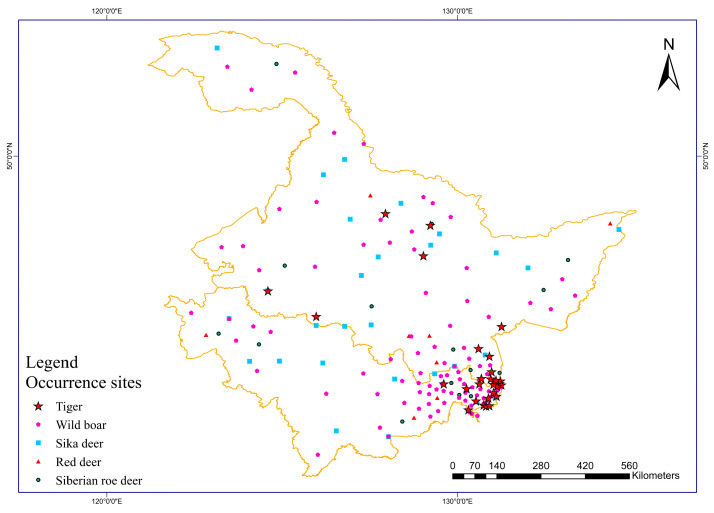
The occurrence sites of the Amur tigers and four ungulates.

Deer (Cervidae) species and wild boar are the main prey of the tiger [24]. The Global Biodiversity Information Facility (GBIF; https://www.gbif.org/, accessed on 5 January 2023) is the world’s largest biodiversity resource library. The species distribution information in this database comes from different channels, such as museum specimens, field observations, and the literature. This study obtained the distribution data of the prey of Amur tigers from the GBIF [25] and established a layer for prey potential richness. The “occ_search” function in R was used to retrieve the distribution records of the four main prey species of the Amur tiger, Siberian roe deer, wild boar, red deer, and Sika deer, from the GBIF website. We carefully checked and eliminated problematic distribution records from GBIF [26], including distribution points with latitude and longitude = 0, duplicate record points, distribution points falling into the ocean, and uncertain record points. We ensured that these observation records occurred between 2015 and 2020. Then, the “CoordinateCleaner” package [27] was used to filter the data to ensure that only one ungulate species of the same species appears within each 1 km grid (Figure 2).

Global climate data with a resolution of 5 min (WorldClim, http://www.worldclim.org/bioclim, accessed on 5 January 2023) and the vegetation factor data (Normalized Difference Vegetation Index (NDVI), https://ecocast.arc.nasa.gov/data/pub/gimms/, accessed on 5 January 2023) were used as a predictive variable for biological variables. These data present average values from 1970 to 2000. Data with resolutions of 30 s were selected and obtained using the raster package get Data function. Combined with the “appearance” and “random absence” data of ungulates mentioned above, the Random Forest package [28] was used to model the potential richness of the main prey of the Amur tiger for the first time. Random forests are a combination of tree predictors such that each tree depends on the values of a random vector sampled independently and with the same distribution for all trees in the forest. The generalization error for forests converges to a limit as the number of trees in the forest becomes large. The generalization error of a forest of tree classifiers depends on the strength of the individual trees in the forest and the correlation between them [28]. For background data, it is common to compare the value of the predicted variable with the observed position of an animal with the value of the unobserved position of that animal [29]. If a species has no preference for any predictive variable (or other variables that are not in the model but are related to the predictive variable), the “random expectation” (also known as “background” or “random missing” data) of the species will be obtained. We used the sample random function in the “raster” function package to select 200 “random missing” data points throughout the entire study area.

Based on the first established random forest regression model, we ranked the importance of each variable and then used “% IncMSE” or “increase in average variance” as an indicator to determine the importance of the predicted variables. This indicator represents the increase in the model prediction error after randomly assigning and replacing each prediction variable; therefore, a larger value indicates a greater importance of the variable. By performing ten-fold cross-validation, the bioclimate prediction variables were selected based on the cross-validation curve. The role of the cross-validation method is to attempt to use different training/validation set partitions to train/validate the model in multiple sets to address the question of single test results being one-sided and insufficient training data. The training set was used for cross-validation. Based on the cross-validation results, the variables that entered the model were selected, and these variables were used to construct a random forest model to obtain the prey potential richness prediction layer [29].

#### 2.2.2. Prediction of Suitable Habitats for the Amur Tigers

##### Obtaining Distribution Data for Amur Tigers

The distribution data of Amur tigers were obtained from the GBIF database and field surveys. The method for obtaining GBIF data is detailed in Section 2.2.1. The field survey data mainly comes from the occurrence point information of the Amur tiger, which was obtained from the 2020 to 2022 transect surveys, infrared camera detection, and interview surveys. After screening and elimination, it was ensured that there was only one occurrence point of the Amur tiger in each 1 km grid. Ultimately, a total of 29 valid records were obtained (Figure 2). The sample random function was used in the “raster” package to generate data on the “appearance” and “random missing” of the Amur tiger. The specific method is described in Section 2.2.1. Fifty “random missing” data points were selected from the entire study area.

##### Environmental Variables

The environmental variables included terrain, vegetation, human interference, and climatic factors. The terrain factor data included the altitude, slope, and aspect. The altitude data were obtained from SRTM (Shuttle Radar Topography Mission, https://earthexplorer.usgs.gov/, accessed on 5 January 2023). A digital elevation model with a spatial resolution of 90 m was obtained. The slope and aspect data were calculated using the ArcGIS10.6 spatial analysis tool. The vegetation factor data were from the Normalized Difference Vegetation Index (NDVI). The NDVI has a linear or nearly linear relationship with green leaf density, photosynthetic effective radiation, vegetation productivity, and cumulative biomass. It is an important parameter that reflects surface vegetation growth and nutritional information and is recognized as an effective indicator of large-scale surface vegetation coverage and growth status. NDVI data were obtained from the GIMMS NDVI3g v1.0 dataset (https://ecocast.arc.nasa.gov/data/pub/gimms/, accessed on 5 January 2023). A spatial resolution of 8 km and a temporal resolution of 15 days were used for monthly maximum value synthesis (MVC) using the GIMMS packages, resulting in monthly maximum value synthesis data from 1982 to 2015. Human interference factor data using the annual dynamic dataset of the Global Land Human Footprint Map from 2000 to 2018 (https://doi.org/10.6084/m9.figshare.16571064, accessed on 5 January 2023) were used to reflect the intensity of human activity [30], which was developed using eight variables from 2000 to 2018, including building environment, population density, night-time lighting, farmland, pasture, highways, railways, and navigable waterways, which reflect different aspects of human stress. Continuous annual human footprint data are crucial for monitoring human stress and conducting research on species extinction risk, conservation science, and human development potential. Climate data use a 30 s resolution of global climate data (World-Clim, please refer to: http://www.worldclim.org/bioclim, accessed on 5 January 2023) and Section 2.2.1 for the acquisition method.

In summary, there were a total of 27 potential variables that affected the distribution of Amur tigers. All of the above variable layers were resampled to a 1 km grid size by bilinear interpolation.

##### Model Fitting and Testing

Based on the random forest algorithm, the environmental variable data, prey potential richness prediction data, and “appearance” and “random absence” data of the Amur tiger in Section 2.2.2 were modeled. The Random Forest package [28] was used to fit the model. Based on the already constructed random forest regression model, we ranked the importance of each variable using the “Mean Decrease Accuracy” as an indicator. This indicator represents the degree to which the accuracy of random forest prediction is reduced by changing the value of a variable to a random number. The larger the value, the greater the importance of the variable. By performing ten-fold cross-validation, variables were selected based on the cross-validation curve and the importance ranking results of the variables. The variable screening method is described in detail in Section 2.2.1.

The ROC curve (area under the curve (AUC) of the subject’s operating curve) was used to assess the performance of the model. The AUC was defined as the area under the ROC curve. The AUC can be obtained by summing the areas of each part of the ROC curve [31]. The AUC values ranged from 0 to 1. The criteria for judging the performance of a classifier (prediction model) based on AUC were as follows: AUC = 1, perfect; AUC = [0.85, 0.95], the effect is very good; AUC = [0.7, 0.85], the effect is average; AUC = [0.5, 0.7], with lower effectiveness; AUC = 0.5, similar to random guessing; the model has no predictive value; AUC < 0.5, worse than random guessing [32]. The data were split into a training set (75%) and a testing set (25%), and the ROC curve was used to test the actual forest model obtained from the classification variables (“appearing” and “randomly missing” sites) on both datasets, extracting prediction probabilities and outputting ROC curve. The true skill statistic (TSS) takes into account both omission and commission errors and success as a result of random guessing and ranges from −1 to +1, where +1 indicates perfect agreement and values of zero or less indicate a performance no better than random [33]. The optimal threshold point was determined using the Youden index, also known as the correct index, which refers to the sum of sensitivity + specificity − 1 (Youden index = Sensitivity + Specificity − 1). The prediction probability was converted into a prediction classification, and the confusion matrix and other indicators were output. Finally, the model was used to predict suitable habitats for Amur tigers in the northeast region. The prediction function was used to obtain a suitable habitat map for the Amur tigers. All the above operations were implemented in R3.3.4.

## 3. Results

### 3.1. Prediction of Prey Potential Richness in Amur Tigers

**Figure 3:**
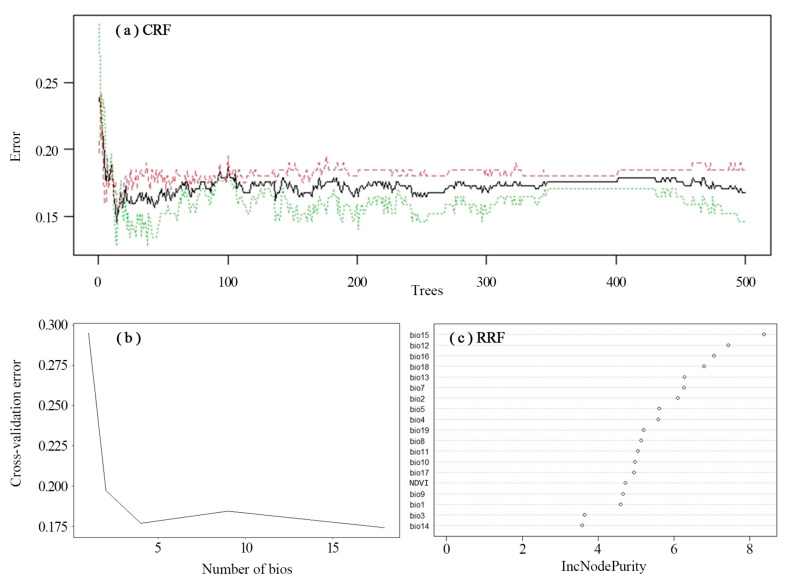
(**a**) Generalization error rate of the prey model for the presence/absence situation. The black, red, and green lines represent all observations, occurrences, and random non-occurrences that vary with the number of decision trees, respectively. (**b**) Cross-validation curve of variables in the Amur tiger prey potential richness model. (**c**) Dot chart of random forest variables importance of prey.

A total of 164 effective records of Amur tiger prey were obtained after screening and eliminating record points. By examining the relationship between the number of decision trees and the generalization error rate (OOB error rate), the red, black, and green lines represent the changes in the error rate of all the samples, prey (ungulate species) occurrence points, and random missing points, respectively, with the number of decision trees. As the number of decision trees in the forest increased, the misjudgment rate rapidly decreased and gradually stabilized after a period of fluctuation. From the graph, it can also be seen that when the decision tree was approximately 20, the overall misjudgment rate was the lowest. However, the result was not stable at this time, and there were some fluctuations afterward, which became relatively stable after more than 500 trees. When the number of decision trees was 500, the overall misjudgment rate of the model was the lowest and tended to stabilize (Figure 3a). Thus, 500 was chosen as the number of decision trees. The cross-validation curve of the relationship between the prey model error and the number of bioclimate variables used for fitting showed a relationship between the model error and the number of variables used for fitting; the error decreased to the lowest when the number of variables was 4 and 19 (Figure 3b).

According to the dot chart of random forest variable importance (Figure 3c), four important climate factor variables were retained: annual precipitation (bio 12), seasonal precipitation (bio 15), precipitation in the wettest region (bio 16), and precipitation in the warmest region (bio 18).

**Figure 4:**
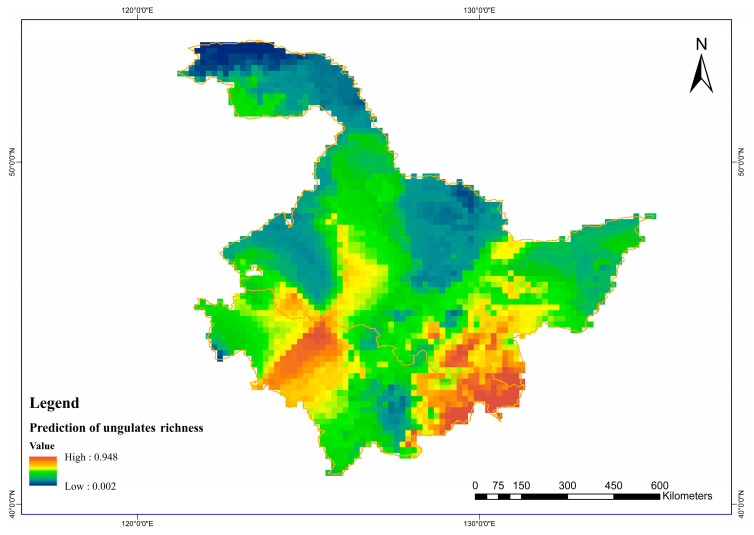
Prediction of prey potential richness in the Amur tigers.

This model was used to predict the potential richness of ungulates in the study areas and generate continuous spatial predictions. From the predicted results, it can be seen that the areas with high potential richness of prey for the Amur tiger were mainly concentrated in the southern border areas of the Laoyeling Mountains between Jilin and Heilongjiang provinces, as well as in most areas of the central and southern Northeast Plain. The potential richness of Amur tiger prey in the Greater and Lesser Khingan Mountains was relatively low. The potential richness of Amur tiger prey in the Sanjiang Plain and the Wanda Mountains was moderate (Figure 4).

### 3.2. Prediction of Suitable Habitats for Amur Tigers

**Figure 5:**
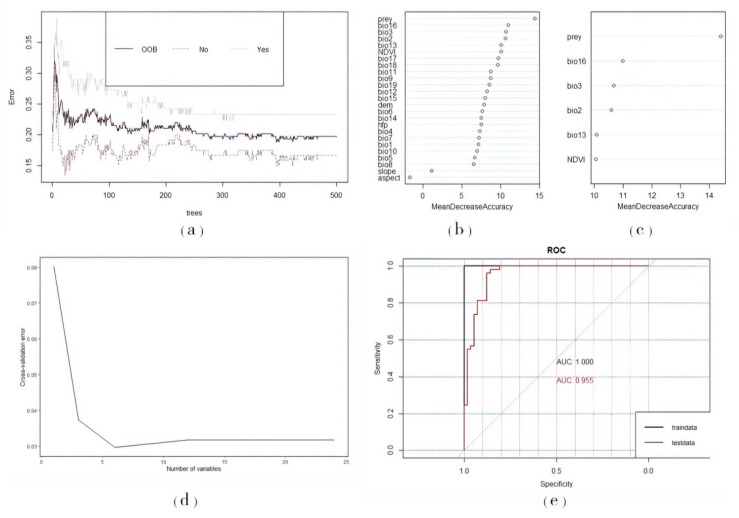
(**a**) Generalization error rate of the Amur tiger model for presence/absence situations. (**b**) Importance ranking of variables in the analysis of suitable habitat selection for Amur tigers. (**c**) Variables with the top six importance rankings. (**d**) The importance of cross-validation curves in the analysis of suitable habitat selection for Amur tigers. (**e**) AUC under ROC curve.

After screening and eliminating the record points of the Amur tigers, 29 valid records were obtained. First, all 27 variables were used to establish a random forest model and evaluate the importance of the variables. By examining the relationship between the generalization error rate (OBB error rate) and the number of random trees, it was found that when the number of decision trees was 500, the overall misjudgment rate of the model was the lowest and tended to stabilize (Figure 5a). Thus, 500 decision trees were selected for this study. By ranking the importance of variables (Figure 5b), it can be seen that the prey variable has the highest importance, whereas the slope and aspect variables of the terrain factors have lower importance. The cross-validation curve shows the relationship between the model error and the number of variables used for fitting. The error first decreased with an increase in the number of variables, and the initial decrease was significant. When the number of variables was increased to six, the error decreased to the lowest point and then increased slightly. After ten-fold cross-validation, according to the cross-validation curve, the error was minimized when six important variables were retained, and the model prediction achieved the desired results (Figure 5d). The top six variables were selected and ranked according to the importance of the Mean Decrease Accuracy variable for the model. Based on their importance, they were prey potential richness (prey), precipitation in the wettest region (bio 16), isotherm (bio 3), daily average range (bio 2), precipitation in the wettest month (bio 13), and normalized vegetation index (NDVI) from high to low (Figure 5c).

The data were split into a training set (75%) and a testing set (25%) for model validation to obtain the AUC values and ROC curves. The results showed that the AUC value of the test set was 0.955 (Figure 5e). The true skill statistic (TSS) value is 0.5924, indicating that the model had good prediction accuracy.

**Figure 6:**
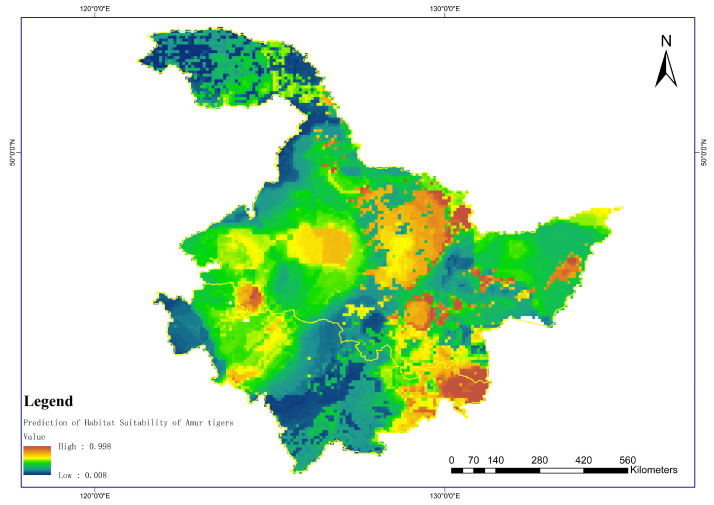
Prediction of habitat suitability of Amur tigers.

According to the prediction results obtained from the model, the areas with high habitat suitability for the Amur tiger were mainly concentrated in the Hunchun–Suiyang Sino-Russian border areas in the southern part of the Laoyeling Mountains, which borders Jilin and Heilongjiang provinces. The site of occurrence of Amur tigers in the Sino-Russian border areas of Raohe County in the eastern part of the Wanda Mountains was relatively low, but their suitability was also high. Although there were no occurrence sites for Amur tigers in the Lesser Khingan Mountains region, their habitat suitability was relatively high. The suitability of habitats in the plain areas connecting Harbin City and Changchun City was relatively low, whereas the suitability in the Greater Khingan Mountains was relatively low (Figure 6).

### 3.3. Comparison of Potential Habitat Characteristics between Amur Tigers and Their Prey

**Figure 7:**
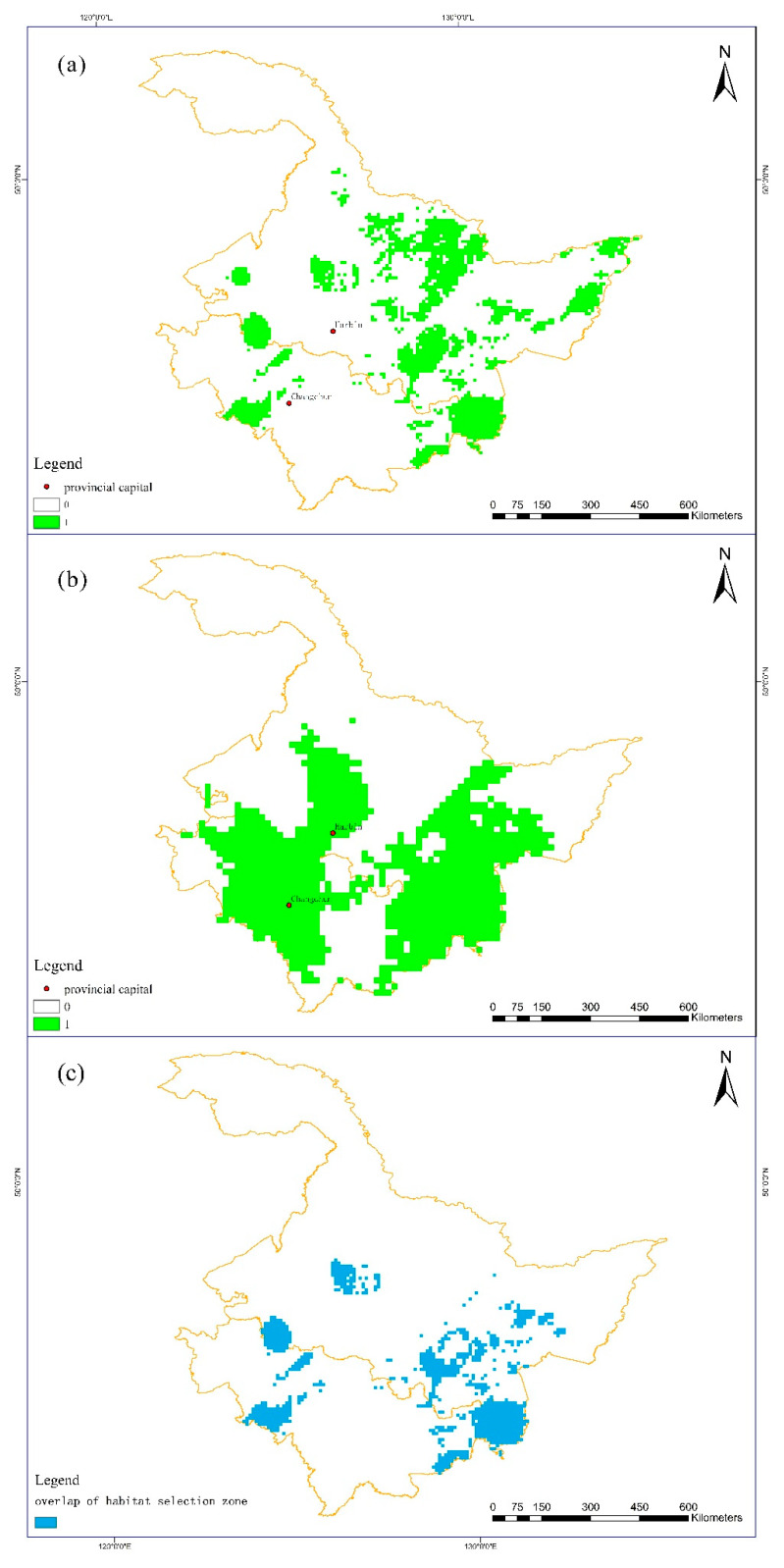
(**a**) Prediction of Amur tiger habitat selection. (**b**) Prediction of Amur tiger prey occupancy selection. (**c**) Overlap of habitat selection zone.

Using the optimal threshold determined by the Youden index as the cutoff value, prediction maps of habitat selection for Amur tigers (Figure 7a) and prey habitat selection for Amur tigers (Figure 7b) were obtained. The results showed that the habitat selected by Amur tiger prey was 249,600 km^2^, accounting for 37.8% of the total study area. The habitat chosen by Amur tigers was approximately 107,600 km^2^, accounting for 16.3% of the total study area. The overlapping areas accounted for 8.52% of the total areas. The habitat chosen by the Amur tiger for prey was concentrated in the Changbai Mountain areas at the border of the eastern Heilongjiang–Jilin provinces and most of the Songnen Plain. The selectivity of the Greater and Lesser Khingan Mountains areas, as well as the Sanjiang Plain and Wanda Mountains areas, was relatively low. The preferred habitat for the Amur tiger was mainly concentrated in the Hunchun–Suiyang Sino-Russian border areas in the southern part of the Laoyeling Mountains, which borders Jilin and Heilongjiang provinces, as well as the Raohe County—Russian border areas in the eastern part of the Wanda Mountains. Small areas were also selected in the Lesser Khingan Mountains. The habitat selection of the Amur tiger prey presented two large areas in the north and south, and there was a connection between the two. Compared with its prey, the habitat chosen by the Amur tiger had a small total area, scattered distribution, low continuity, and a lack of connectivity between patches.

## 4. Discussion

A study comparing the results of five machine learning models (classification tree, random forest, artificial neural network, support vector machine, and automatically induced rule-based fuzzy model) found that random forest performed better [34]. Random forests establish a relationship between several independent variables, X, and the dependent variable, Y, by generating a large number of classification trees. The learning process for the random forest was fast. However, it is still efficient in processing large amounts of data. Existing random forest algorithms evaluate the importance of all variables without worrying about the multicollinearity problem faced by general regression problems. This includes algorithms for estimating missing values that can maintain a certain degree of accuracy if a portion of the data is lost [32]. This study used data on the occurrence points of Amur tiger prey to predict the potential richness of Amur tiger prey and then used these data and other environmental factors to predict the suitable habitat for Amur tigers. Research has found that prey potential richness is the most critical factor driving the distribution of Amur tigers, which is consistent with the existing research results. This further proves that sufficient prey resources in existing studies are the foundation and prerequisite for effective recovery and protection of the Amur tiger population [4].

The potential suitable habitat for the Amur tiger is approximately 107,600 km^2^, covering 16.3% of the total study areas; it is mainly distributed in the southern part of the Laoyeling Mountains (Hunchun–Wangqing–Dongning–Suiyang) at the border between Jilin and Heilongjiang provinces, along the border with Russia (within the Amur tiger and Leopard National Park). It is located in the southern part of the Laoyeling Mountains, a tributary of Changbai Mountain, and is mainly characterized by medium to low mountains, canyons, and hills. The highest elevation is 1477.4 m (Laoyeling Mountains), belonging to the continental humid monsoon climate zone with a forest coverage rate of 93.32%. The vegetation type is mainly temperate coniferous broad-leaved mixed forest, which is the core area for the distribution of wild Amur tigers in China and borders the distribution areas of Russian Amur tigers, which is conducive to the cross-border activities of Amur tigers. Although there are limited data on the distribution sites of Amur tigers in the Sino-Russian border areas of Raohe County in the eastern part of the Wanda Mountains, their habitat suitability is relatively high. In addition, the habitat suitability of the Lesser Khingan Mountains in Yichun Forest Areas is relatively high. The suitability of habitats in the central part of the study areas is relatively low, and the areas with high development intensity of construction land in the northeast region are concentrated in the two major urban agglomerations in the northeast region: the Harbin–Changchun City Group and the Central and Southern Liaoning City Group [35]. This situation may be caused by the dense highways, railways, and residential areas between Harbin and Jilin, as well as the high intensity of human activity. The banded distribution of road networks and other human facilities may cause fragmentation of suitable habitats for Amur tigers and fragmentation of habitat patches. The traffic flow of provincial highways is large, which has a great impact on the Amur tiger ecological corridor, and the expressway has a greater impact on the Amur tiger ecological corridor [36]. The low connectivity and accessibility of habitat landscapes may have a negative impact on the migration of Amur tigers. Forest coverage in the Greater Khingan Mountains was high, but its suitability was low, which may be related to the lack of data on the prey and vegetation types of the Amur tiger in the Greater Khingan Mountains. However, this may also be related to differences in climate and vegetation types between the Greater Khingan Mountains and the Lesser Khingan Mountains. The high vegetation coverage in the Greater Khingan Mountains may inhibit the activity of Amur tigers or increase hunting difficulties.

Owing to the lack of survey data on prey density, this study included only the predicted potential richness data as a variable in the model. However, the number of prey is a basic requirement for the survival of Amur tigers, and the prey density should not be less than 0.5 ungulates/km^2^; otherwise, female tigers cannot litter and feed them [2]. Therefore, the potential habitat simulated in this study may have overestimated the actual suitable habitat for the Amur tigers. From these results, it can be seen that prey is the most important factor affecting the distribution of Amur tigers. On the border of the Laoyeling Mountains between the two provinces of Heilongjiang–Jilin and Russia, areas with higher prey potential richness overlapped with suitable habitats for the Amur tiger; both indices in the Greater Khingan Mountains were relatively low. However, the potential richness of prey in the eastern region of the Wanda Mountains and Lesser Khingan Mountains was relatively low, but the habitat suitability of the Amur tiger was relatively high, indicating that prey is not the only factor affecting the distribution of Amur tigers. The eastern part of the Wanda Mountains is a temperate southern coniferous broad-leaved mixed forest zone with a dense water system. The Yichun Forest areas of the Lesser Khingan Mountains are the most concentrated and well-preserved core areas of broad-leaved Korean pine (*Pinus koraiensis*) forests in China [37,38], with Korean pine as the dominant species of zonal vegetation, accompanied by various broad-leaved trees. This may provide better food, water, shelter, and other necessary survival conditions for Amur tigers, and further research is needed. Compared with their prey, the habitat chosen by Amur tigers had a small total area, severe fragmentation, and a lack of connectivity between regions. This suggested that some factors may restrict the diffusion of the Amur tiger, whereas the diffusion of ungulates is less restricted. However, the reason for this phenomenon requires further investigation.

Due to the availability of data, the time between the tiger occurrence data (2020–2022), the vegetation data (1982–2015), and human population density (2000–2018) do not match. Therefore, there is probably a bias in the development of the models.

## 5. Conclusions

The suitable habitat for the Amur tiger was mainly distributed in the southern part of the Laoyeling Mountains (Hunchun–Wangqing–Dongning–Suiyang) at the border between Jilin and Heilongjiang provinces, Huilin–Raohe areas of the Sino-Russian border in the eastern part of the Wanda Mountains, and Yichun Forest areas in the Lesser Khingan Mountains. The suitability of the Greater Khingan Mountain region was relatively low. Compared to other factors, prey potential richness has a greater impact on the distribution of Amur tigers; however, prey was not the only factor affecting the distribution of Amur tigers. Further research is needed to support the optimization of the natural reserves of Amur tigers, which have high habitat requirements. The potential suitable habitat of Amur tigers in the study was small and severely fragmented, with a lack of connectivity between patches. The results suggested that habitat protection, restoration, and ecological corridor construction should be strengthened to increase population dispersal and exchange, providing a reference for the conservation of the Amur tiger in the future.

## Data Availability

Data are contained within the article.
